# Prenatal diagnosis and genetic counseling of an inherited unbalanced chromosome abnormalities in a Chinese family

**DOI:** 10.1186/s13039-022-00614-0

**Published:** 2022-08-15

**Authors:** Ying Zhang, Juan Chen, Zonghui Feng, Wencheng Li

**Affiliations:** 1 Reproductive Medicine Center, Renmin Hospital, Hubei University of Medicine, Shiyan, Hubei People’s Republic of China; 2 Prenatal Diagnosis Center, Renmin Hospital, Hubei University of Medicine, Shiyan, Hubei People’s Republic of China; 3 Hubei Clinical Research Center for Reproductive Medicine, Shiyan, Hubei People’s Republic of China; 4 Biomedical Engineering College, Hubei University of Medicine, Shiyan, Hubei People’s Republic of China; 5 Prenatal Diagnosis Center, Maternity and child Care Hospital of Huaihua, Huaihua, Hunan People’s Republic of China

**Keywords:** Chromosomal microarray analysis (CMA), Noninvasive prenatal testing (NIPT), Chromosomal microdeletions/microduplications, Prenatal diagnosis, Unbalanced chromosomal abnormalities (UBCA)

## Abstract

**Background:**

Unbalanced chromosome abnormalities (UBCA) are either gains or losses or large genomic regions, but the affected person is not or only minimally clinically affected. Copy number variants (CNVs) are an important source of normal and pathogenic genome variations. CNVs and UBCA identified in prenatal cases need careful considerations and correct interpretation if those are harmless or harmful variants from the norm.

**Case presentation:**

A 25-year-old, gravida 1, para 0, woman underwent amniocentesis at 18 weeks of gestation because the noninvasive prenatal testing (NIPT) results revealed a 6.8 Mb duplication from 2q11.1 to 2q11.2. Chromosomal microarray analysis (CMA) was performed on uncultured amniocytes. GTG-banding karyotype analysis on cultured amniocytes was performed.

**Results:**

Chromosomal GTG-banding of the cultured amniocytes revealed a karyotype of 46,XX. CMA detected a 6.8-Mb chromosomal duplication in the region of 2q11.1q11.2 (arr[GRCh37] 2q11.1q11.2(95,327,873_102,088,148)x3).

**Conclusion:**

Chromosomal microdeletions and microduplications are difficult to detect by conventional cytogenetics, combination of prenatal ultrasound, karyotype analysis, NIPT, CMA and genetic counseling is helpful for the prenatal diagnosis of UBCA and chromosomal microdeletions/microduplications.

## Introduction

Noninvasive prenatal testing (NIPT) is widely used in the screening of common fetal chromosome aneuploidy [1]. Conventional karyotyping provides an overview of the entire genome and can identify structural and numerical chromosome abnormalities. Chromosomal microarray analysis (CMA) is a method using array technology to detect chromosome abnormalities spanning less than 5 Mb [2].

Unbalanced chromosomal abnormalities (UBCA) were reported for euchromatic regions of many human autosomes. Carriers of UBCA are in many cases clinically healthy, and UBCA are often nothing else than cytogenetically visible copy number variants (CNVs) [3, 4].

Because CMA does not require cell culture, samples which cannot be cultured by conventional karyotyping can be analyzed with CMA, and CMA offers faster testing result. However, conventional karyotyping is limited to detect the rearrangement with a length longer than 5 Mb, which can be detected by CMA [5] and CMA cannot detect balanced translocations, which can be detected by conventional karyotyping [6].

Here we report the prenatal diagnosis and genetic counseling of a maternally inherited chromosome 2q11.1q11.2 duplication in a Chinese family with normal phenotype using NIPT, chromosomal GTG-banding and CMA.

## Methods

### Patients and samples

A 25-year-old, gravida 1, para 0, woman underwent amniocentesis at 18 weeks of gestation because the noninvasive prenatal testing (NIPT) results revealed 6.8 Mb duplication from 2q11.1 to 2q11.2. Her husband was 25-year old too. There was no family history of birth defects or genetic diseases. GTG-banding karyotype analysis was performed on cultured amniocytes and parental blood samples. CMA on uncultured amniocytes was performed using the Affymetrix CytoScan 750 K chip, which includes 550k non-polymorphic markers and 200k SNP markers.

## Results

**Fig. 1:**
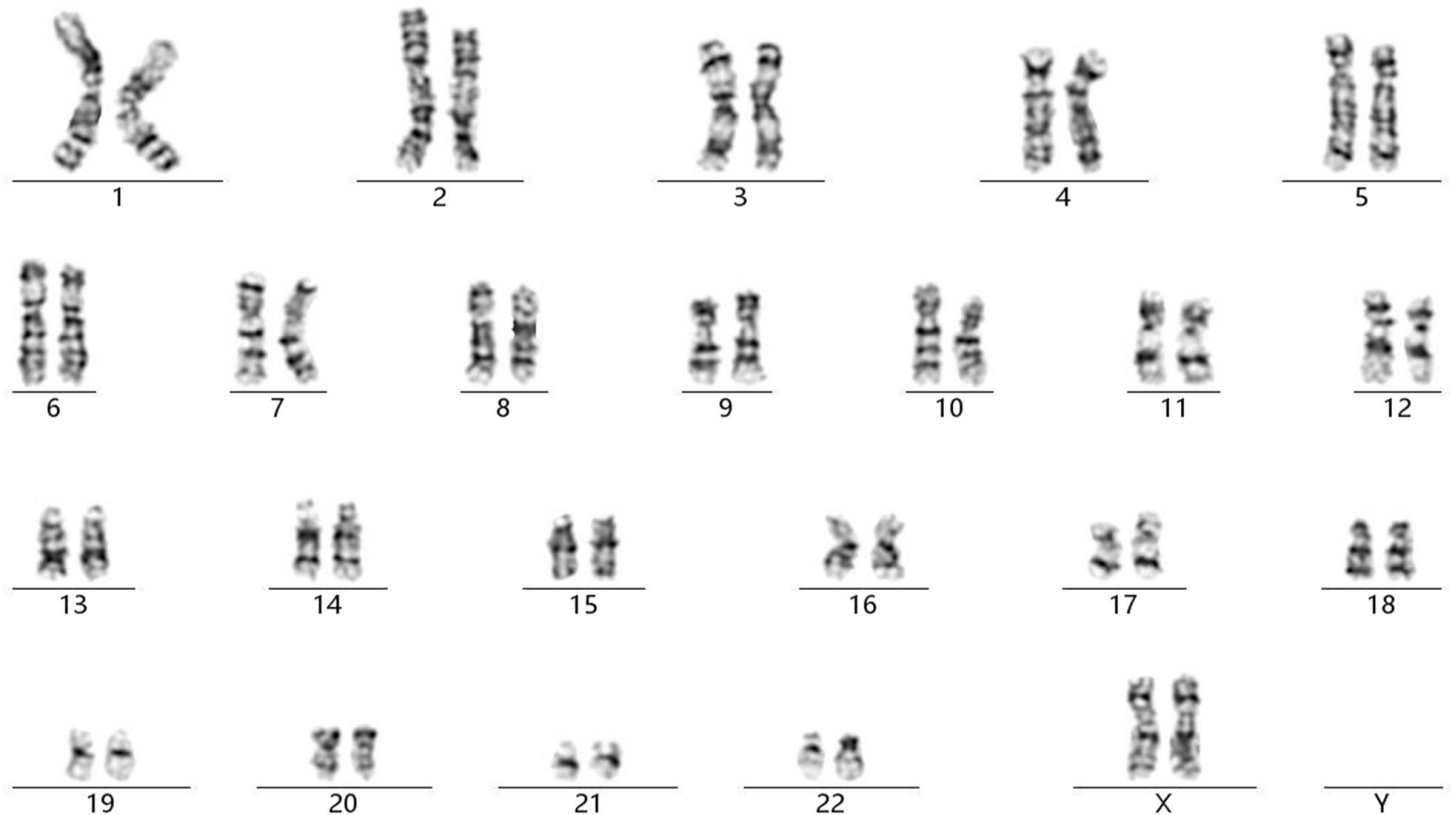
The karyotype of 46,XX

**Fig. 2:**
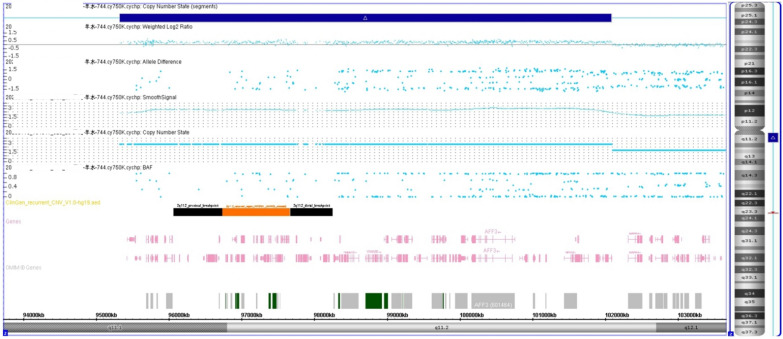
CMA detected a 6.8-Mb chromosomal duplication in the region of 2q11.1q11.2 (arr[GRCh37] 2q11.1q11.2(95,327,873_102,088,148)x3)

**Fig. 3:**
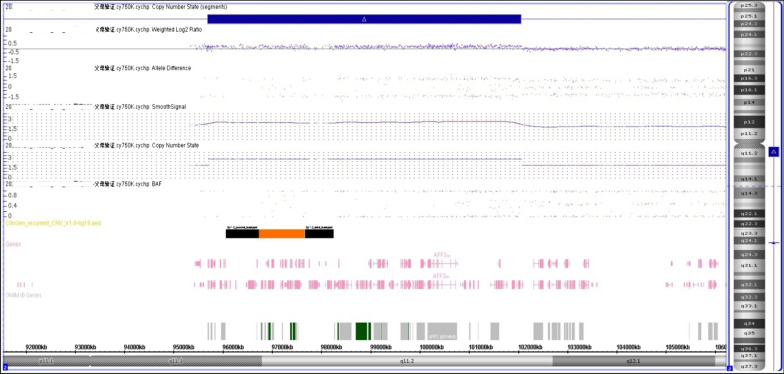
CMA detected a 6.4-Mb chromosomal duplication in the region of 2q11.1q11.2 (arr[GRCh37] 2q11.1q11.2(95,694,601_102,064,543)x3)

Chromosomal GTG-banding revealed a karyotype of 46,XX (Fig. 1). CMA detected a 6.8-Mb chromosomal duplication in the region of 2q11.1q11.2, which is to be reported according to International System of Cytogenomic Nomenclature 2020 (ISCN 2020) [7] as arr[GRCh37] 2q11.1q11.2(95,327,873_102,088,148)x3 (Fig. 2). Then we performed both CMA and chromosomal GTG-banding using the samples from the parents’ peripheral blood. Their karyotypes were normal. The CMA results showed the mother had a 6.4-Mb chromosomal duplication -- arr[GRCh37] 2q11.1q11.2(95,694,601_102,064,543)x3 like the fetus (Fig. 3). We performed a comprehensive physical examination of the parents and failed to identify anything abnormal.

Ultrasound examination showed no dysmorphisms or intrauterine growth restriction (IUGR) in the fetus. After genetic counseling, the parents decided to continue the pregnancy.

At 39 weeks of gestation, the expectant mother gave birth vaginally to a female baby. The baby’s growth parameters at birth were in the normal ranges. Apgar scores were 9/9/10. The baby received a complete physical examination and the results were normal. At 36-month checkup, the baby was developing normally (Intelligence Quotient, IQ = 107).

## Discussion

In this study, the chromosomal duplication of 2q11.1q11.2 contains several genes, just as *ARID5A* and *LMAN2L*, and these genes are all triplo-insensitive genes.

Our observation is in agreement with the uncritical region for centromere-near gain of copy numbers of chromosome 2 as defined by sSMCs [8]. According to the literature [9–11] yet only several cases/ families with partial trisomies of chromosome 2q11.1q11.2 are reported, which did not show any or minimal clinical signs. In literature 8, an unbalanced karyotype, 46,XX,der(8),ins(8;2)(p21.3;q21.1qll.2), was found in the proband and her mother, who both have mild mental retardation, short stature, dysmorphic features, insulin dependent diabetes mellitus, and a psychotic illness. This family is a rare example of direct transmission of a partial autosomal trisomy. In our study, the mother and her baby both have the duplication of 2q11.1q11.2, and they both have normal phenotype.

Predicting the phenotypic outcome of prenatally diagnosed duplication of 2q11.1q11.2 remains challenging. Important efforts have been devoted to define the effects of duplication of 2q11.1q11.2, but the available information is scarce.

During pregnancy, there were no dysmorphisms or IUGR in the fetus. At the 3-year follow-up, the baby did not have an abnormal phenotype and exhibited no evidence of developmental delay. This observation provided credence to the concept that trisomies of 2q11.1q11.2 may not contribute to abnormal phenotype. However, further study is needed to understand the pathogenic affect of 2q11.1q11.2 trisomies. We plan to follow this patient and her mother in order to monitor their phenotype.

NIPT is a very efficient and accurate method for the detection of chromosome aneuploidy. Recently, further expansion of NIPT through deeper sequencing has focused on additional screening for microdeletion and microduplications, which had also notable screening results [1]. CMA is superior to standard karyotype in detection of chromosomal microdeletion/microduplication [12]. But in another aspect, we highlights the necessity to be careful in hasty conclusions about the potential impact of gains or losses as detected in NIPT or CMA analyses. Without a parental genetic test and best also a GTG-banding the nature and impact of a detected imbalance cannot be interpreted reliably.

## Conclusion

Combination of prenatal ultrasound, karyotype analysis, NIPT, CMA and genetic counseling is helpful for the prenatal diagnosis of UBCA and chromosomal microdeletions/microduplications.

Herein the case of a (sub)chromosomal imbalance expressed as duplication of 2q11.1q11.2 is presented, which is per definition an UBCA without obvious clinical consequences for two carriers within the same family. The case highlights that prenatal detection of even large CNVs implicates parental testing to come to a well-funded estimation on the impact of the identified alteration [13, 14].

## Data Availability

Please contact the corresponding author for data requests.
